# Severely Obese Adolescents and Adults Exhibit a Different Association of Circulating Levels of Adipokines and Leukocyte Expression of the Related Receptors with Insulin Resistance

**DOI:** 10.1155/2013/565967

**Published:** 2013-12-21

**Authors:** Antonello E. Rigamonti, Fiorenza Agosti, Alessandra De Col, Giancarlo Silvestri, Nicoletta Marazzi, Silvia Bini, Sara Bonomo, Marialuisa Giunta, Silvano G. Cella, Alessandro Sartorio

**Affiliations:** ^1^Department of Clinical Sciences and Community Health, University of Milan, Via Vanvitelli 32, 20129 Milan, Italy; ^2^Istituto Auxologico Italiano, IRCCS, Experimental Laboratory for Auxo-Endocrinological Research, Via Ariosto 13, 20145 Milan, Italy; ^3^Istituto Auxologico Italiano, IRCCS, Division of Metabolic Diseases, Corso Mameli, 199, 28921 Verbania, Italy

## Abstract

Obese adults frequently exhibit a low-grade inflammation and insulin resistance, which have been hypothesized to be established early in childhood. Aim of this study was to evaluate the age-dependent relationships between inflammatory state and insulin resistance in obese adolescents and adults. Clinical and metabolic parameters, circulating adipokines (TNF-**α**, adiponectin, and leptin), ghrelin, their leukocyte receptors (TNFR1, ADIPOR2, OBRL and GHSR1a), and acute phase reactants (CRP and white blood cells) were assessed in lean and obese adolescents compared with the adult counterparts. Only obese adults had higher HOMA-IR, insulin, and triglycerides compared to the lean group. An inflammatory state was present in obese adolescents and adults, as demonstrated by the higher values of CRP and neutrophils. There were no group differences in circulating levels of TNF-**α** and leukocyte expression of TNFR1. Adiponectin concentrations and leukocyte expression of ADIPOR2 were higher in the lean groups than in the corresponding obese counterparts. For leptin and leukocyte expression of OBRL, the results were opposed. Circulating levels of ghrelin were higher in lean adolescents and adults than the related lean groups, while there was a higher leukocyte expression of GHSR1a in (only) lean adults than obese adults. When the analysis was performed in (lean or obese) adults, TNF-**α**, neutrophils, leptin, and GHSR1a were predictors of HOMA-IR. None of the considered independent variables accounted for the degree of insulin resistance in the adolescent group. In conclusion, a dissociation between the low-grade inflammation and insulin resistance is supposed to exist in the early phases of obesity.

## 1. Introduction

Obesity is considered a low-grade chronic inflammatory disease that may contribute to the development of insulin resistance [1]. Obese adolescents and adults are at higher risk for developing type 2 diabetes (T2D), cardiovascular disease, nonalcoholic fatty liver disease (NAFLD), osteoarticular diseases, and several forms of cancer [2].

Recent clinical studies have suggested that mediators of low-grade chronic inflammation, such as adipokines, cytokines, ghrelin, and acute phase reactants, are involved in the development of these comorbid conditions [3, 4].

Tumor necrosis factor-*α* (TNF-*α*) is one of the main mediators of the inflammatory response in obesity and is expressed by infiltrating macrophages and adipocytes in the hypertrophic adipose tissue [5]. TNF-*α* receptors 1 (TNFR1) and 2 (TNFR2) are the two main transducers of the TNF-*α* signals in most cells and tissues, including peripheral leukocytes [6].

Adiponectin, an adipose tissue secreted protein, has been well recognized to exhibit insulin-sensitizing, anti-inflammatory, and antiatherosclerotic properties, which are mediated through its receptors, ADIPOR1 and ADIPOR2 [7, 8]. These receptors are ubiquitously expressed in most organs as well as in peripheral monocytes and in monocyte-derived macrophages [9–15]. Adiponectin levels are known to be decreased in patients with obesity, T2D, and coronary artery disease (CAD), promoting the establishment of an inflammatory state in these conditions [16–19].

Leptin is another adipocyte-secreted hormone that centrally regulates food intake and body weight [20]. Obese subjects may become resistant to leptin resulting in greater production and secretion of this peptide [21]. Leptin is correlated with insulin resistance and is considered a key mediator linking insulin resistance with metabolic syndrome and NAFLD [22]. The leptin receptor is expressed not only in the central nervous system, but also in peripheral tissues, such as haematopoietic and immune systems contributing to the obesity-related inflammatory state [23]. The leptin receptor (OBR) is present in several alternative splice variants, of which only the long isoform (OBRL) is fully functional [24, 25].

Ghrelin is a stomach-derived hormone that, differently from leptin, stimulates food intake and growth hormone (GH) release [26]. Circulating levels of ghrelin are reduced in obesity [27] and metabolic syndrome [28], and, among obese subjects, ghrelin has been reported to be lower in insulin-resistant patients [29]. The effects of ghrelin are mediated via specific receptors, named the GH secretagogue receptors (GHSR) [30]. Two distinct ghrelin receptor transcripts are known: GHSR type 1a acts as ghrelin receptor, whereas type 1b is a truncated form [31]. The GHSR1a is mainly expressed in the hypothalamus and pituitary gland [32], but it has also been found in many peripheral organs, including immune cells, in which ghrelin inhibits different proinflammatory functions [33–35].

NAFLD, which frequently occurs in obesity, is the consequence of excessive accumulation of triglycerides by hepatocytes. Acute phase reactants produced in the liver, such as C-reactive protein (CRP) and fibrinogen, are elevated in obese adults and are implicated in the development of cardiovascular disease and T2D [36–38].

Obese adults, who have a long-term duration of the disease, frequently exhibit a low-grade inflammation and insulin resistance, which have been hypothesized to be dissociated in childhood [39]. However, there is not complete consensus on this matter in the paediatric literature [40, 41].

We hypothesized that the relationship between key biomarkers of obesity and inflammation in adolescents may be established in an initial (and hopefully reversible) phase. To test this hypothesis, we have assessed clinical and metabolic parameters, pro- and anti-inflammatory adipokines (TNF-*α*, adiponectin, and leptin) and ghrelin together with their receptors (TNFR1, ADIPOR2, OBRL, and GHSR1a) and acute phase reactants (CRP and white blood cells), in a group of lean and obese adolescents compared with the adult counterparts.

## 2. Materials and Methods

### 2.1. Subjects

Obese male adolescents (age: 15.0 ± 0.5 yrs; body mass index (BMI): 40.3 ± 2.2 kg/m²) and adults (age: 36.5 ± 1.9 yrs; BMI: 45.4 ± 1.3 kg/m²) were recruited from the Istituto Auxologico Italiano, IRCCS, Verbania, Italy, where they were admitted for a multidisciplinary body weight-reduction intervention. All obese adults suffered from overweight and obesity since childhood. Exclusion criteria included previous diagnosis of any disease affecting the endocrine system and metabolism such as T2D, chronic use of medications affecting metabolism and/or appetite, and ≥5.0 kg weight change during the 3 months preceding the study participation. Healthy lean male adolescents (age: 14.3 ± 0.1 yrs; BMI: 20.5 ± 0.5 kg/m²) and adults (age: 32.1 ± 2.7 yrs; BMI: 24.1 ± 0.8 kg/m²) were recruited among friends/colleagues and their sons. Tanner stage was evaluated in all (lean and obese) adolescents with no intergroup difference.

The four experimental groups (obese and lean adolescents and adults) were socioeconomically homogenous when evaluated by a specific questionnaire [42].

The study was approved by the local Ethic Committee. All subjects participated in this study after providing their free and informed consent. For subjects aged less than 18 yrs, the informed consent was provided by their parents.

### 2.2. Anthropometric Measurements

Before blood sampling, height and weight were measured with the subject standing in light clothes and without shoes. The BMI was calculated as weight/height^2^ (kg/m^2^). Waist and hip circumferences were also calculated. All examinations were performed by the same investigator.

### 2.3. Blood Collection

Following a 10–12-hour fast, blood was collected from all participants at 09:00 hours in a standardized fashion. Standard blood tests, such as white blood cell (WBC) count with differential, comprehensive metabolic profile including glucose, cholesterol, triglycerides, and insulin levels and CRP, were performed at the Clinical Laboratories of the Istituto Auxologico Italiano, IRCCS, Verbania, Italy. In addition to the blood samples used for the above-reported standard blood tests, a blood sample (about 40 mL) was drawn and placed immediately on ice. Plasma and serum were obtained after centrifugation and kept at −80°C for biochemical measurements (precisely TNF-*α*, leptin, adiponectin, and ghrelin), while mononuclear cells were isolated by using a Vacutainer CPTTM (Becton Dickinson, NJ, USA), a sterile cell preparation tube with sodium citrate. Cells recovered from the layer just under the plasma layer were washed twice with PBS pH 7.4 and, after a last centrifugation, the pellet was suspended again by using an adapted amount of a specific buffer, which stabilizes cellular RNA (Ambion United Kingdom, distributed in Italy by Celbio, Milan). This cellular suspension was then stored at −80°C until RNA extraction (see below). Circulating levels of adipokines and ghrelin and leukocyte expression of the related receptors were evaluated at the Neuroendocrinology Labs, Department of Clinical Sciences and Community Health, University of Milan, Milan, Italy.

### 2.4. Estimation of Insulin Resistance

As an estimate of insulin resistance, fasting glucose and insulin levels were used to compute the homeostatic model assessment of insulin resistance (HOMA-IR), which was calculated in accordance with the following formula: (insulin (*μ*IU/mL) × glucose (mg/dL))/405.

### 2.5. RNA Preparation

Total RNA was isolated from the mononuclear cells using the Trizol reagent (Life Technologies, Carlsbad, CA, USA) according to the manufacturer's instructions. After purification RNA was treated with DNase (DNA-free-Ambion) to avoid false-positive results due to amplification of contaminating genomic DNA. Concentrations of total RNA were determined from the absorbance value of the sample at 260 nm. First-strand cDNA was synthesized from 1 *μ*g of total RNA in a final volume of 40 *μ*L using a High-Capacity cDNA Archive kit (Applied Biosystems, Forster City, CA, USA). The Kit uses random primers to initiate cDNA synthesis. The reaction conditions were 25°C for 10 min and 37°C for 2 h.

### 2.6. Real-Time PCR

cDNA (2 *μ*L) was subjected to real-time quantitative PCR using ABI PRISM 7000 (Applied Biosystems, Forster City, CA, USA). TaqMan PCR was performed in 25 *μ*L volumes using TaqMan Universal PCR Master Mix (Applied Biosystems, Forster City, CA, USA). TaqMan probe/primers specific for GAPDH (code no. AX-004253-00-0400), human TNFR1 (code no. AX-003934-00-0200), human ADIPOR2 (code no. AX-007801-00-0200), human OBRL (code no. AX-008015-00-0200), and human GSR1a (code no. AX-005513-00-0200) were purchased from Euroclone, Pero, Italy.

All PCR assays were performed in triplicate and the data were pooled. Before using the ΔΔ*C*_*T*_ method for relative quantification, we performed a validation experiment to demonstrate that the efficiencies of target and reference were approximately equal.

The reaction conditions were as follows: 50°C for 2 min, 95°C for 10 min, followed by 40 cycles at 95°C for 15 s (denaturation) and 60°C for 1 min (annealing and elongation).

As controls, we used the reaction mixtures without the cDNA.

Threshold cycle numbers (*C*_*T*_) were determined with an ABI PRISM 7000 Sequence Detection System (version 1.1 software) and transformed using the Δ*C*_*T*_ (2^−ΔΔ*C*_*T*_^) comparative method. Gene-specific expression values were normalized to expression values of GAPDH (endogenous control) within each sample. The levels of mRNA of each receptor were expressed relative to the calibrator value of the same receptor in the corresponding lean (adolescent or adult) group (controls). Relative quantification was performed using the comparative method. The amount of target, normalized to an endogenous reference and relative to a calibrator, is given by 2^−ΔΔ*C*_*T*_^. Briefly, the Δ*C*_*T*_ value is determined by subtracting the average GAPDH *C*_*T*_ value from the average receptor *C*_*T*_ value in the same sample; the calculation of ΔΔ*C*_*T*_ involves subtraction of the Δ*C*_*T*_ calibrator value.

### 2.7. Biochemical Measurements

Plasma concentrations of adiponectin and leptin were measured by two commercially available radioimmunoassay (RIA) kits (Millipore, St. Charles, MO, USA). For adiponectin the sensitivity of the method was 1 ng/mL; intra- and interassay coefficients of variation (CV) were 1.78–3.59% and 6.90–9.25%, respectively; for leptin the sensitivity of the method was 0.44 ng/mL; intra- and interassay CV were 3.4–8.3% and 3.0–6.2%, respectively.

Plasma TNF-*α* concentrations were measured by an enzyme-linked immunoabsorbent assay (ELISA) kit (IBL International GmbH, Hamburg, Germany). The limit of detection of the method was determined to be 5.0 pg/mL. Intra- and interassay CV were 2.0–14.9% and 4.1–14.0%, respectively.

Total immunoreactive ghrelin concentration was determined with a commercial RIA kit (Millipore, Research Park Drive, St. Charles, MO, USA). The lower and upper detection limits were 93 pg/mL and 6000 pg/mL, respectively, whereas intra- and interassay CV (at 1500 pg/mL) were 3.3% and 17.8%, respectively.

IGF-1 concentrations were determined by the commercially available immunometric chemiluminescence assay Immulite 2000 (DPC; Los Angeles, USA). Intra- and interassay CV were 2.9% and 7.4%, respectively. The sensitivity of the assay was 20 ng/mL.

Plasma glucose, (total) cholesterol, and triglycerides were determined by commercial colorimetric methods (Sigma Diagnostics, St. Louis, MO, USA).

Serum concentrations of insulin were measured by immunometric chemiluminescence (Immulite 2500, DPC; Los Angeles, USA); the sensitivity of the method was 2 *μ*IU/mL; intra-assay and interassay CV were <5.5% and <7.3%, respectively.

A high-sensitivity immunochemiluminescent method was used to measure serum levels of CRP (Immulite 2500, DPC; Los Angeles, USA), the analytical sensitivity being 0.01 mg/dL. The intra-assay and interassay CV were <8.7% for both parameters.

For each biochemical parameter, all of a single subject's samples were run in the same assay and the order of tubes in the assay was randomized.

### 2.8. Statistical Analysis

Data were presented as mean ± SEM (standard mean error).

As conventionally established, the sample size was determined for giving 80% power at the 0.05 level of significance (two-sided). The expected mean difference of one adipokine (precisely, fasting leptin levels, i.d., ~20 ng/mL) between lean and obese groups and the estimated standard deviation of the same variable (i.d., ~3.0 ng/mL) were deducted from the results reported in [20] and on the basis of our personal previous experiences.

The Shapiro-Wilk test was used to test data normality. One-way ANOVA, followed by Bonferroni's test, was used to investigate group differences in demographic, clinical, inflammatory, metabolic, and endocrine parameters. Exploratory analyses of all data were conducted by using Pearson's correlation to investigate whether TNF-*α*, adiponectin, leptin, and ghrelin, their related receptors (TNFR1, ADIPOR2, OBRL, and GHSR1a) acute phase reactants (CRP and white blood cells), and HOMA-IR were associated with all other parameters. To formally test whether levels of the adipokines, ghrelin, their receptors, and inflammatory markers accounted for the degree of insulin resistance, backward stepwise linear regressions were calculated with HOMA-IR as dependent variable and TNF-*α*, adiponectin, leptin, and ghrelin, their related receptors (TNFR1, ADIPOR2, OBRL, and GHSR1a), acute phase reactants (CRP and neutrophil granulocytes), BMI, and age as independent variables in the entire population (lean and obese adolescents and adults) and in the two groups of (lean or obese) adolescents and adults. Data were analyzed using SPSS for Windows version 19.0 (SPSS, Inc., Chicago, IL, USA). A *P* < 0.05 was considered statistically significant.

## 3. Results

Demographic, clinical, inflammatory, metabolic, and endocrine data of the whole population recruited in the study are reported in Table 1.

As expected, obese adults had significantly higher HOMA-IR and fasting insulin and triglycerides values compared to the corresponding lean group. Although not statistically significant, higher HOMA-IR, fasting insulin, and triglycerides values were also found in obese adolescents when compared to those of the corresponding lean group (Table 1).

An inflammatory state was present in obese subjects, both adolescents and adults, as demonstrated by the significant higher values of CRP and neutrophil granulocytes (only absolute number) (Table 1).

**Figure 1:**
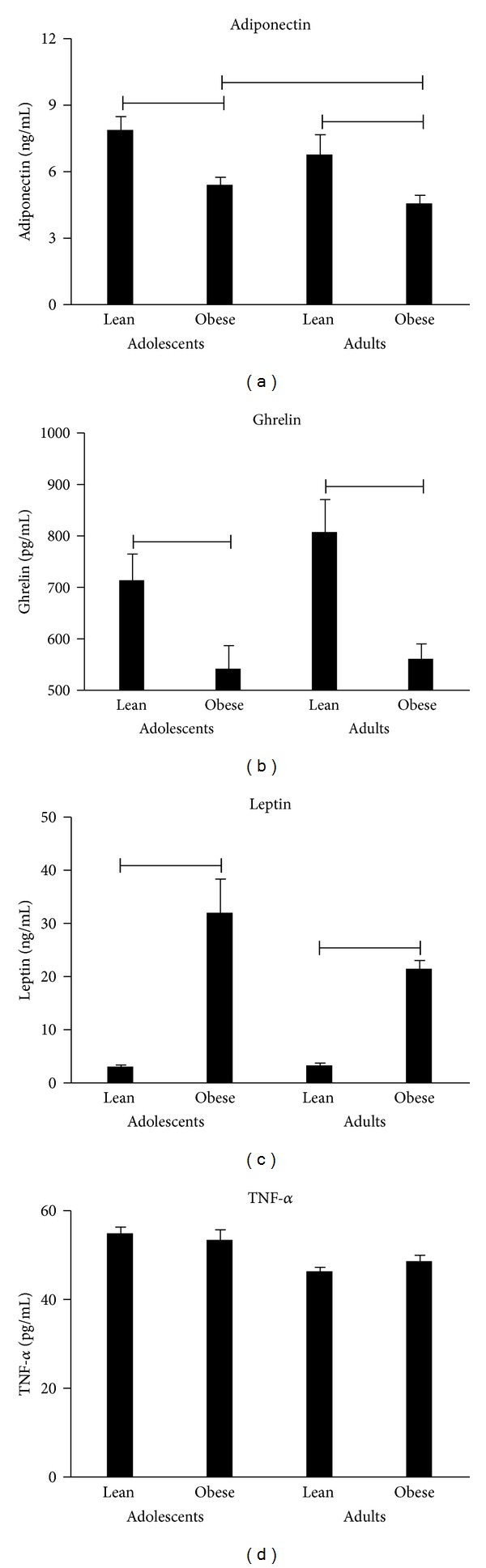
Circulating levels of adiponectin, ghrelin, leptin, and TNF-*α* in lean and obese adolescents and adults. Comparisons: *P* < 0.05.

**Figure 2:**
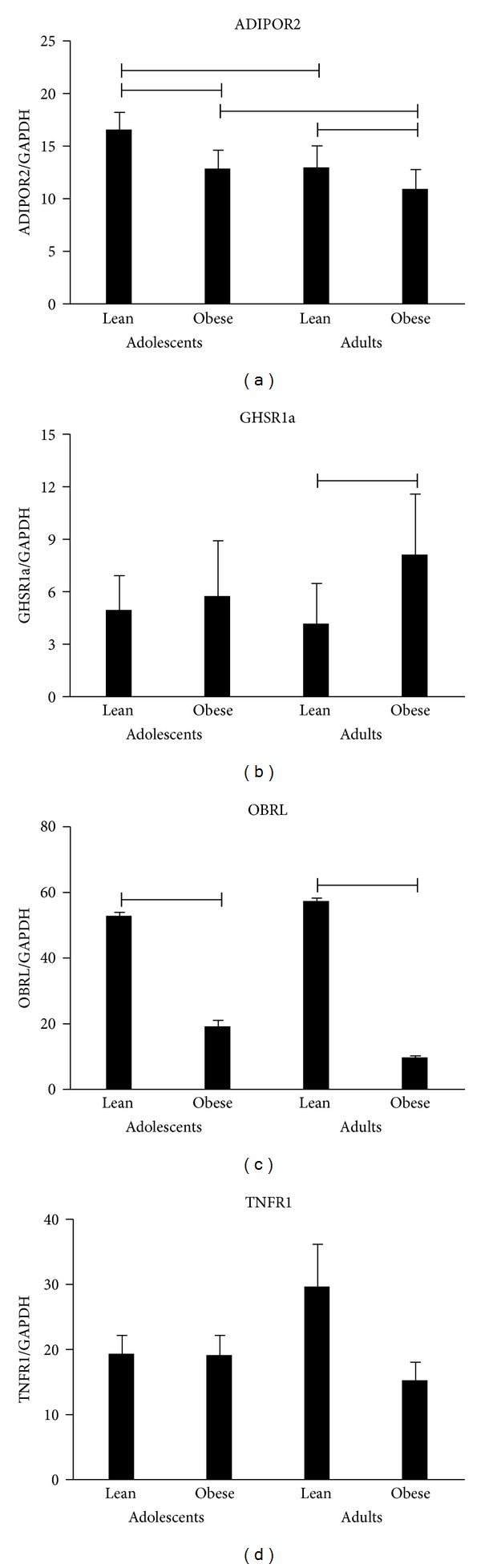
Leukocyte expressions of ADIPOR2, GHSR1a, OBRL, and TNFR1 in lean and obese adolescents and adults. Comparisons: *P* < 0.05.

There were no group differences in circulating levels of TNF-*α* and leukocyte expression of TNFR1 (Figures 1 and 2). Adiponectin concentrations were significantly higher in the lean groups than in the corresponding obese counterparts; interestingly, obese adolescents had higher levels of this adipokine than obese adults (Figure 1). Also leukocyte expression of ADIPOR2 was significantly higher in the lean groups than in the corresponding obese counterparts; irrespective of BMI, adolescents had higher leukocyte mRNA levels of ADIPOR2 than adults (Figure 2). As expected, leptin concentrations were significantly higher in obese (adolescent and adult) groups than in the lean counterparts, whereas there was a lower leukocyte expression of OBRL in obese adolescents or adults than in the corresponding lean groups (Figures 1 and 2). Opposed to these results, circulating levels of ghrelin were significantly higher in lean adolescents and adults than in the related obese groups, while there was a higher leukocyte expression of GHSR1a in (only) lean adults than in obese adults (Figures 1 and 2).

Tables 2–4 summarize all significant correlations of TNF-*α*, adiponectin, leptin, and ghrelin, their related receptors (TNFR1, ADIPOR2, OBRL, and GHSR1a), acute phase reactants (CRP and white blood cells), and HOMA-IR with all other parameters. Among these results there were the correlations of adiponectin with neutrophil granulocytes, ghrelin, and leptin, of ghrelin with insulin, lymphocytes, monocytes, and leptin, of leptin with CRP, insulin, and all leukocyte subpopulations, and of TNF-*α* with IGF-1, neutrophil granulocytes, lymphocytes, and leptin (Table 2). Leukocyte expression of TNFR1 was positively and negatively correlated, respectively, with those of ADIPOR2 and GHSR1a, while circulating levels of leptin were negatively correlated with mRNA levels of OBRL in leukocytes (Table 3). Finally, HOMA-IR was correlated positively with neutrophil granulocytes but negatively with leukocyte expression of ADIPO-R2 (Table 4).

Findings from the above (bivariate) correlation analysis were further explored using the stepwise multiple linear regression analysis with HOMA-IR as a dependent variable. When all data (i.e., lean and obese adolescents and adults) were considered, TNF-*α* (*β* = 0.27, *P* < 0.01) and neutrophil granulocytes (absolute number) (*β* = 0.36, *P* < 0.01) were the only significant predictors of HOMA-IR. When the analysis was performed in (lean or obese) adults, in addition to TNF-*α* (*β* = 0.34, *P* < 0.01) and neutrophil granulocytes (absolute number) (*β* = 0.43, *P* < 0.01), also leptin (*β* = 0.51, *P* < 0.01) and GHSR1a (*β* = 0.41, *P* < 0.01) were significant predictors of HOMA-IR. Interestingly, none of the considered independent variables (i.e., adipokines, ghrelin, their receptors, inflammatory markers, BMI, and age) accounted for the degree of insulin resistance in the adolescent group.

## 4. Discussion

Over the last two decades, our view of adipose tissue has been changed from that of an “inert” storage tissue to an “active” endocrine organ able to secrete a number of adipokines, such as adiponectin, TNF-*α*, and leptin, which are reported to exert potent neuroimmunoendocrine effects [43–45].

Reportedly, obesity promotes a state of chronic low-grade inflammation, which is reflected not only by an increased production of pro-inflammatory cytokines and adipokines by adipose tissue but also by a cellular component, which is represented by macrophages whose number is dramatically increased in obesity. Thus, local synthesis and release of chemoattractants that enhance the homing of monocytes to adipose tissue depots are thought to induce adipose tissue inflammation [46].

The development of T2D is characterised by two processes: (1) insulin resistance, resulting from impaired insulin signalling and leading to an increased demand for insulin, which must be met by increased insulin production by pancreatic *β* cells (compensatory *β*-cell function); (2) *β*-cell dysfunction, with T2D developing when the amount of insulin that is produced is insufficient to meet the demand [47].

The low-grade inflammation found in overweight and obesity, especially in case of abdominal fat accumulation, has been implicated in *β*-cell dysfunction and induction of insulin resistance [1, 48].

Differently from the situation in obese adults, the pathophysiology of inflammation has not been fully studied in obese adolescents and also no clear-cut consensus exists on the temporal establishment of insulin resistance [39–41].

The population recruited in the present study was composed of lean and obese adolescents and adults. It is noteworthy that only obese adults had significantly higher insulin levels and HOMA-IR values than the corresponding lean group, while in obese adolescents the statistical significance was not reached because of a widespread variability of the same metabolic parameters, which is well known in paediatric literature [49, 50].

Most studies in obese paediatric population show consistent increases in CRP, but not in TNF-*α*, which is frequently reported to be elevated in obese adults [5, 39, 51]. In the present study, irrespective of CRP, which was higher in obese adolescents and adults compared to the lean counterparts, there were no group differences in circulating levels of TNF-*α* and leukocyte expression of TNFR1, which is one of the receptors to which this cytokine binds [52]. These findings are not surprising because other works found similar circulating levels of TNF-*α* in lean and obese subjects [53, 54], one reason being the combination of extremely low concentrations of this cytokine and the sensitivity of the analytical method [55]. Anyway, the lack of any group difference in TNF-*α* does not damper the potential pathophysiological role of this cytokine.

In fact, when all data (i.e., lean and obese adolescents and adults) were pooled in the analysis of stepwise linear regression, TNF-*α* and neutrophil granulocytes were significant predictors of HOMA-IR. Interestingly, TNF-*α* and neutrophil granulocytes still accounted for the degree of insulin resistance in the adult but not in the adolescent group. Based on these findings, one might argue that TNF-*α* plays a key role in low-grade inflammation-induced *β*-cell dysfunction that precedes the overt insulin resistance in obese adults [5, 56]. The adolescents recruited in the present study may have been obese for a relatively short time (which corresponds to their young age, i.e., 10–15 years). If inflammatory cytokines are the result of macrophage infiltration into the adipose tissue [46], it is possible that more time is required for this infiltration and the deleterious effects of TNF-*α* to occur. Thus, a dissociation between low-grade inflammation and insulin resistance appears to be present in obese adolescents [39].

This study has confirmed that the long leptin receptor splice variant (OBRL) is expressed in blood mononuclear cells from lean and obese subjects [25]. Obese adolescents and adults had lower relative expression levels of OBRL mRNA transcripts compared with lean counterparts. Interestingly, there was an overall significant inverse correlation of leptin receptor transcript with the BMI and the circulating leptin levels, suggesting that the degree of adiposity and/or the elevation in leptin levels might influence the gene expression of OBRL.

Assuming that the downregulation of OBRL reflects a similar reduction in leptin receptor numbers on the cell surface (i.e., mRNA versus protein levels), this might contribute to the apparent leptin resistance of obese individuals. In support of this intriguing hypothesis, there is experimental evidence to suggest that the expression of leptin receptor(s) is sensitive to both genetic and physiologic interventions that cause a change in circulating leptin levels [57, 58]. In this context, ob/ob mice that lack leptin have elevated levels of total leptin receptor expression [57].

In the present study, there was no difference in leukocyte expression of OBRL between obese adolescents and adults, this finding being expected because of the similar high leptin levels in both obese groups. While HOMA-IR was not associated with OBRL, leptin was a predictor of HOMA-IR only in obese adults. Therefore, the downregulation of OBRL in obese subjects seems to be only a consequence of the high leptin levels, whereas leptin should be considered a determinant factor in worsening of insulin resistance in obese adults. This is congruent with the well-known association of hyperleptinemia with insulin resistance [59]. The correlations of leptin with CRP and white blood cells, which were found also in the present study, confirm the implication of this peptide in sustaining the low-grade inflammation and, ultimately, *β*-cell dysfunction in obesity [56]. Anyway, these correlations could also be due to any other metabolic factors related to hyperleptinemia actually present in obesity.

Ghrelin has been reported to promote GH release and regulate energy metabolism [60]. Furthermore, ghrelin may play a role in energy balance, glucose and lipid metabolism, and blood pressure regulation [61]. These physiological functions can explain the associations of low serum ghrelin levels with insulin resistance, T2D, and blood hypertension in general population [62] as well as carotid atherosclerosis in older people with metabolic syndrome [63–65]. Although there are few studies in animal models and humans to indicate that ghrelin has any anti-inflammatory effect [66], progression of the low-grade inflammation in obesity might be accelerated by decrease in circulating levels of ghrelin and/or GHSR1a-mediated signalling in immune cells. In the present study, as expected, obese subjects had significantly lower levels of ghrelin than the corresponding lean groups; anyway, only obese adults exhibited an upregulation of GHSR1a in leukocytes. While ghrelin was negatively correlated with insulin, leukocyte expressions of GHSR1a and TNFR1 were inversely correlated. Importantly, GHSR1a was a predictive factor of HOMA-IR in obese adults, but not in obese adolescents. These findings would suggest the establishment of a (deleterious?) compensatory mechanism mediated by GHSR1a in obese adults to overcome the reduced levels of ghrelin, an allegedly diabetogenic peptide [67].

Adiponectin is a versatile cardiovascular protective factor, which plays an important role in regulating insulin sensitivity and energy homeostasis, with antiinflammatory and anti-atherosclerotic properties [7]. Gene expression of this adipokine is downregulated in both obesity and T2D [68]. Hypoadiponectinemia is an independent risk factor for CAD in T2D [69]. Under pathological conditions, including obesity and T2D, hypoxia, oxidative stress, and inflammation suppress adiponectin secretion from adipose tissue [70, 71]. Interestingly, exogenous supplementation of recombinant adiponectin attenuates insulin resistance, improving metabolic disorders [69].

In the present study, circulating levels of adiponectin were lower in obese subjects compared to the lean groups; furthermore, obese (but not lean) adolescents had higher adiponectin levels than the corresponding adult group. As no age-dependence of adiponectin levels was found, a persistent obese state might be involved in the reduced secretion of this adipokine in the adult age or, alternatively, the markedly low adiponectin levels might explain the hyperinsulinemia and the higher HOMA-IR values in obese adults when compared to the obese adolescents.

Leukocyte expressions of ADIPOR2 were significantly higher in the lean groups than in the corresponding obese counterparts, indicating an obesity-related downregulation of this receptor, which, combined with the hypoadiponectinemia, might explain the establishment of insulin resistance in obese adults. Along this view, the findings that adiponectin and ADIPOR2 were negatively correlated, respectively, with white blood cells and HOMA-IR would suggest the existence of a link among adiponectin signalling, low-grade inflammation, and insulin resistance [69].

In accordance with the negative correlation of ADIPOR2 with age, (lean or obese) adolescents had higher leukocyte mRNA levels of this receptor than (lean or obese) adults. Furthermore, the correlations of ADIPOR2 with leptin, and TNFR1 or of adiponectin with ghrelin and leptin would indicate a crosstalk among adipokines and the related receptors, although other metabolic factors might be decisive for altered secretion of ghrelin, leptin and adiponectin as well as for the altered expression of ADIPOR2 and TNFR1 [72]. Alterations of a single component of the adipokine system would provoke deleterious effects at systemic levels, particularly on glucose metabolism [73].

In the present study, many correlations of adipokines, ghrelin, and the related receptors with anthropometric and metabolic parameters were found. With these associations being well known in the literature, no further discussion is necessary. Anyway, some correlations, including those of GHSR1a with height or of IGF-1 with TNF-*α*, appear to be of great interest, but their discussion is beyond the aims of the present study.

Before closing, some limitations of the present research need to be mentioned. First of all, only a limited number of adipokines/cytokines/peptides and related receptors were investigated; anyway, the adipokine profiling that was defined in the present study is sufficient to suppose the existence of a dissociation between insulin resistance and low-grade inflammation in obese adolescents and of an unbalance of pro- and anti-inflammatory factors in obese adults, who are at high risk to develop insulin resistance and T2D. Second, receptor expression was evaluated in leukocytes, which are easily isolated from whole blood; anyway, biopsy specimens from adipose tissue or hypothalamus, which would have provided more information, represent an invasive or impracticable approach; the notion that leukocytes include monocytes from which adipose macrophages are derived should be recalled. Third, the results should be cautiously interpreted because they are only based on statistical considerations, which do not actually imply any form of causation. Finally, the present study is cross-sectional and only two distinctive obese (not-diabetic) groups (i.e., adolescents and adults) were recruited. A long-term prospective study, including obese adolescents with at least 20-year followup, would allow us to better evaluate the role of the single adipokines and the receptor changes in the pathophysiology of insulin resistance and T2D, but this is, obviously, difficult to conduct not only for economic reasons.

In conclusion, this study demonstrates that obese adults exhibit alterations in circulating levels of some adipokines (particularly, adiponectin and leptin) and ghrelin and in leukocyte expressions of the related receptors (i.e., ADIPOR2, OBRL, and GHSR1a), which sustain a low-grade chronic inflammation as demonstrated by their associations with some acute phase reactants, including CRP and white blood cells. In the same group TNF-*α*, leptin, GHSR1a, and neutrophil granulocytes are predictive factors of HOMA-IR. Since none of the adipokines tested, ghrelin, the related receptors, and acute phase reactants are associated with HOMA-IR in obese adolescents, a dissociation between the low-grade inflammation and insulin resistance is supposed to exist in the early phases of obesity.

Based on these remarks, any effort, including diet, exercise, and pharmacological intervention, should be made in paediatric obese population to early contrast the low-grade inflammation and to avoid the occurring of an overt insulin resistance. Novel pharmacological strategies, including adipokine agonist/antagonists or anti-inflammatory drugs, might be of valuable interest in this context [72, 74].

## Figures and Tables

**Table 1:** Demographic, clinical, metabolic, haematological, and hormonal characteristics of the study population.

	Adolescents		Adults	
	Lean	Obese	Lean	Obese
No.	19	17	15	15
Age (yrs)	14.3 ± 0.1^b,c^	15.0 ± 0.5	32.1 ± 2.7	36.5 ± 1.9
Weight (kg)	60.2 ± 1.9^a,c^	116.4 ± 8.2	74.9 ± 2.4^a^	139.9 ± 5.0
Height (m)	1.7 ± 0.0	1.7 ± 0.0^b^	1.8 ± 0.0	1.8 ± 0.0
BMI (kg/m^2^)	20.5 ± 0.5^a,c^	40.3 ± 2.2^b^	24.1 ± 0.8^a^	45.4 ± 1.3
Waist circumference (cm)	79.2 ± 1.2^a,c^	119.9 ± 5.1^b,c^	85.5 ± 2.7^a^	134.8 ± 2.4
Hip circumference (cm)	94.1 ± 1.2^a,c^	124.4 ± 4.6^b^	97.4 ± 1.6^a^	132.0 ± 4.2
Total cholesterol (mg/dL)	152.7 ± 6.2^b,c^	150.2 ± 4.8^b,c^	187.1 ± 12.1	194.1 ± 12.0
Triglycerides (mg/dL)	70.8 ± 4.5^c^	93.0 ± 6.9^c^	81.1 ± 13.5^a^	152.8 ± 19.8
Glucose (mg/dL)	86.4 ± 1.4	82.2 ± 1.6	96.7 ± 2.3	97.6 ± 8.2
Insulin (*µ*IU/mL)	7.2 ± 1.3	11.9 ± 1.7^b^	4.1 ± 0.7^a^	12.0 ± 1.4
HOMA-IR	1.6 ± 0.3^c^	2.4 ± 0.3^b^	1.0 ± 0.2^a^	3.1 ± 0.6
CRP (mg/dL)	0.0 ± 0.0^a,c^	1.0 ± 0.3^b^	0.1 ± 0.0^a^	0.6 ± 0.1
IGF-1 (ng/mL)	457.2 ± 24.1^a,b,c^	357.9 ± 28.8^b,c^	192.6 ± 21.6	119.9 ± 14.2
Neutrophils (%)	44.8 ± 1.9	51.8 ± 2.6	52.6 ± 2.4	52.5 ± 1.8
Lymphocytes (%)	41.2 ± 1.6	35.2 ± 2.2	34.9 ± 2.3	36.1 ± 1.8
Monocytes (%)	8.1 ± 0.5	8.8 ± 0.4	8.2 ± 0.5	7.7 ± 0.2
Eosinophils (%)	5.3 ± 0.9	3.7 ± 0.5	3.8 ± 0.6	3.3 ± 0.5
Basophils (%)	0.6 ± 0.0	0.5 ± 0.1	0.6 ± 0.1	0.5 ± 0.1
Neutrophils (10^9^/L)	2.8 ± 0.2^a,c^	4.6 ± 0.5^b^	3.2 ± 0.3^a^	4.4 ± 0.4
Lymphocytes (10^9^/L)	2.5 ± 0.1^a^	3.0 ± 0.3	2.2 ± 0.2^a^	2.9 ± 0.1
Monocytes (10^9^/L)	0.5 ± 0.0	0.7 ± 0.1	0.5 ± 0.0	0.6 ± 0.0
Eosinophils (10^9^/L)	0.3 ± 0.1	0.3 ± 0.0	0.4 ± 0.2	0.3 ± 0.0
Basophils (10^9^/L)	0.0 ± 0.0	0.0 ± 0.0	0.0 ± 0.0	0.0 ± 0.0

^a^ *P* < 0.05 versus the corresponding (adolescent or adult) obese group.

^b^ *P* < 0.05 versus the lean adult group.

^c^ *P* < 0.05 versus the obese adult group.

**Table 2:** Correlations of adipokines and ghrelin with all other parameters investigated in the study. Only significant correlations with the respective *r* coefficient are reported (*P* < 0.05).

Adiponectin	
Height	0,227
BMI	−0,232
Total cholesterol	0,216
Insulin	−0,257
Eosinophils %	0,207
Neutrophils *N*.	−0,250
Monocytes *N*.	−0,241
Leptin	−0,317
Ghrelin	0,334
Ghrelin	
Weight	−0,379
BMI	−0,376
Waist circumference	−0,369
Hip circumference	−0,366
Insulin	−0,377
Lymphocytes *N*.	−0,352
Monocytes *N*.	−0,404
Leptin	−0,318
Leptin	
Weight	0,681
BMI	0,750
Waist circumference	0,716
Hip circumference	0,721
Triglycerides	0,292
CRP	0,478
Insulin	0,356
Eosinophils %	−0,240
Neutrophils *N*.	0,542
Lymphocytes *N*.	0,524
Monocytes *N*.	0,569
Basophils *N*.	0,242
TNF-*α*	
Age	−0,375
Total cholesterol	−0,257
Triglycerides	−0,217
Glucose	−0,322
IGF-1	0,413
Neutrophils %	−0,210
Lymphocytes *N*.	0,225
Leptin	0,275

*N*.: absolute number (10^9^/L).

**Table 3:** Correlations of adipokine and ghrelin receptors with all other parameters investigated in the study. Only significant correlations with the respective *r* coefficient are reported (*P* < 0.05).

ADIPOR2	
Age	−0,249
Waist circumference	−0,285
Triglycerides	−0,243
Leptin	−0,255
TNFR1	0,359
GHSR1a	
Height	−0,335
TNFR1	−0,324
OBRL	
Weight	−0,429
BMI	−0,400
Waist circumference	−0,431
Hip circumference	−0,456
Leptin	−0,270
TNFR1	
/	

*N*.: absolute number (10^9^/L).

/: the fact that TNFR1 has no correlation with the other parameters.

**Table 4:** Correlations of inflammatory markers and insulin resistance with all other parameters investigated in the study. Only significant correlations with the respective *r* coefficient are reported (*P* < 0.05).

CRP	
Weight	0,411
Height	−0,279
BMI	0,506
Waist circumference	0,472
Hip circumference	0,471
Neutrophils *N*.	
Weight	0,563
BMI	0,602
Waist circumference	0,576
Hip circumference	0,580
CRP	0,469
Insulin	0,388
Neutrophils %	0,766
Lymphocytes %	−0,747
Eosinophils %	−0,375
Lymphocytes *N*.	
Weight	0,321
BMI	0,362
Waist circumference	0,333
Hip circumference	0,323
Insulin	0,249
Neutrophils %	−0,266
Lymphocytes %	0,315
Neutrophils *N*.	0,343
HOMA-IR	
Total cholesterol	0,228
Neutrophils %	−0,259
Lymphocytes %	0,341
Neutrophils *N*.	−0,298
Eosinophils *N*.	−0,258
ADIPOR2	−0,286

*N*.: absolute number (10^9^/L).

## Abbreviations

ADIPOR2: Adiponectin receptor 2
BMI: Body mass index
CAD: Coronary artery disease
CRP: C-reactive protein
GH: Growth hormone
GHSR1a: GH secretagogue receptor 1a
GHSR1b: GH secretagogue receptor 1b
HOMA-IR: Homeostatic model assessment of insulin resistance
OBRL: Leptin rector (long variant)
T2D: Diabetes mellitus type 2
TNF-*α*: Tumor necrosis factor *α*
TNFR1: TNF-*α* receptor 1
TNFR2: TNF-*α* receptor 2.
